# Teaching clinical communication skills through virtual patient-based learning: an umbrella review of systematic reviews

**DOI:** 10.1186/s12909-026-09374-6

**Published:** 2026-05-13

**Authors:** Matthias Kalmring, Sabine Chmelar, Philipp Greimel, Manuel Kaider, Christoph Lang, Benjamin Roszipal

**Affiliations:** 1 Institute of Health Sciences, Department of Health Sciences, Faculty of Social and Health Sciences, USTP - University of Applied Sciences St. Pölten, Sankt Pölten, Austria; 2 Department of Physiotherapy, Department of Health Sciences, Faculty of Health and Social Sciences, USTP - University of Applied Sciences St. Pölten, Sankt Pölten, Austria; 3 Department Healthcare and Nursing, Department of Health Sciences, Faculty of Health and Social Sciences, USTP - University of Applied Sciences, St. Pölten, Sankt Pölten, Austria; 4 Division of Neonatology, Pediatric Intensive Care and Neuropediatrics, Department of Pediatrics, Comprehensive Center for Pediatrics, Medical University of Vienna, Vienna, Austria

**Keywords:** Clinical communication, Immersive technologies, Virtual patients, Learning, Healthcare students, Medical students, Education

## Abstract

**Background:**

Effective clinical communication is a core component of patient-centered care. Immersive learning technologies, including virtual patients, virtual reality, and augmented reality, are increasingly used to train clinical communication skills in undergraduate health professions education. However, the existing evidence is fragmented and methodologically heterogeneous. This umbrella review synthesizes review-level evidence on immersive technologies and virtual patients for developing clinical communication skills in undergraduate health professions education and explores barriers to curricular integration and key research gaps.

**Methods:**

An umbrella review of systematic reviews (with or without meta-analysis) was conducted following PRISMA guidelines. Searches were performed in PubMed, ScienceDirect, CINAHL, and Cochrane Library. Eligible reviews examined immersive technologies (e.g., VR, AR, MR, XR, virtual patients) targeting clinical communication skills in undergraduate learners. Methodological quality was assessed using AMSTAR-2.

No pooled meta-analysis was feasible because of substantial heterogeneity in interventions, comparison conditions, and outcome measures.

**Results:**

Nine systematic reviews were included. All were rated as critically low in methodological quality, which limits confidence in the conclusions that can be drawn. Across reviews, immersive technologies were reported as potentially beneficial for short-term communication performance, learner engagement, and perceived confidence, particularly in comparison with no intervention or some traditional teaching formats. However, evidence regarding long-term retention, empathy development, non-verbal communication, and transfer to clinical practice remained inconsistent. Substantial heterogeneity in intervention design, outcome measures, and feedback structures limited comparability across reviews.

**Supplementary Information:**

The online version contains supplementary material available at 10.1186/s12909-026-09374-6.

## Background

Communication is a non-linear process in which verbal and nonverbal information is actively exchanged between sender and receiver [1]. It requires core skills such as active listening, empathy, trust-building, and clarity, expressed through spoken language, writing, or body language [2]. In healthcare, clinical communication refers to a structured, empathic, and patient-centered framework that supports information gathering and health-related decision-making in a manner that is understandable and accessible to patients [3, 4]. Despite broad agreement on its importance, clinical communication can be understood as a multifaceted construct that is conceptualized and operationalized differently across educational and clinical contexts [3–5].

Over recent decades, increased attention has been paid to patient education, with strong clinical communication skills now widely recognized as essential for achieving effective, patient-centered care and improved treatment outcomes. Within this context, psychosocial factors, such as cultural background, and patients’ broader lived experiences, play a crucial role in shaping clinical communication quality and, when adequately addressed are associated with higher patient satisfaction and health outcomes [6–8]. However, the integration of these psychosocial dimensions into clinical communication training varies considerably, and such communication skills remain insufficiently developed across healthcare professions [9–12]. To address these gaps, a variety of instructional approaches, including workshops, lectures, and standardized patient encounters have been implemented. Nevertheless, substantial heterogeneity persists in intervention design, instructional intensity, and reporting quality, limiting reproducibility and comparability across studies [5, 13, 14]. In this context, immersive learning technologies such as virtual patients, augmented reality (AR), virtual reality (VR), and mixed reality (MR) have gained attention for their potential to create interactive learning environments and to support clinical communication training through realism, presence, and repeated practice opportunities [15–17]. Low-immersion virtual patients typically offer structured communication scenarios [18, 19], whereas AR or MR integrate digital elements into real-world settings [20] and high-immersion VR provides three-dimensional environments that may enhance learner engagement [15]. However, existing reviews report a fragmented and heterogeneous evidence base, characterized by variability in intervention design, feedback mechanisms, and curricular integration, as well as limited evidence on long-term retention and transfer to clinical practice [13, 15, 16, 21]. This heterogeneity constrains the interpretability of findings and hinders evidence-informed curricular decision-making, underscoring the need for a higher-level synthesis of review-level evidence.

To date, no umbrella review has synthesized the existing systematic reviews on immersive learning approaches on clinical communication skills training in undergraduate healthcare professions education. This umbrella review therefore aims to synthesize the available review-level evidence on immersive learning methods for fostering clinical communication skills among undergraduate learners and to identify implementation-related challenges. By consolidating the current review-level evidence, this work seeks to inform curriculum development and guide future research in digitally supported clinical communication education.

## Methods

Umbrella reviews provide a comprehensive, narrative synthesis of evidence by consolidating findings from systematic reviews and meta-analyses addressing specific topics [22]. This umbrella review was conducted in accordance with the Preferred Reporting Items for Systematic Reviews and Meta-Analyses (PRISMA) guidelines [23] and was preregistered in the Open Science Framework (OSF)(10.17605/OSF.IO/EJ7XB). All methods described in this manuscript were conducted in accordance with the final, updated version of the OSF-registered protocol.

Eligibility criteria were defined using the PICO framework. Included were students in healthcare professions education, with no restrictions regarding age, sex, or geographic location. Systematic reviews, with or without meta-analyses, were eligible if they investigated immersive and virtual patient learning technologies, specifically VR, AR, MR or extended reality (XR), and technology-based simulations, aimed at enhancing clinical communication skills. No restrictions were applied regarding comparison conditions (e.g., control conditions or alternative teaching approaches). Therefore, systematic reviews without a comparison group were also included, which limits the certainty with which relative effectiveness can be inferred.

Clinical communication skills were conceptualized as an umbrella term encompassing both practical abilities and contextual dimensions of patient-provider interaction. For the purposes of this review, this includes constructs such as empathy, understanding of patient perspective, and the formulation of appropriate questions within patient-centered assessment [18]. Reviews were eligible if they reported at least one outcome related to clinical communication, irrespective of whether this outcome constituted the primary focus of the review. Reviews were excluded if they addressed communication outcomes exclusively in other domains, such as interprofessional, leadership, or organizational communication. Only articles published in peer-reviewed journals were included to ensure a robust evidence base. Given that English is the predominant language in high-impact scientific publishing and higher education [24, 25], only English-language publications were considered to ensure methodological consistency and comparability of findings. In light of evidence suggesting that approximately half of systematic reviews become outdated within 5.5 years [26]; the initial search was restricted to publications published between December 31, 2019, and December 31, 2024. An update search was conducted on August 1, 2025, to ensure that the evidence base remained current.

### Search strategy

In March 2025, a systematic literature search was conducted by the lead author in PubMed, ScienceDirect, CINAHL, and the Cochrane Library. Key Concepts and their respective synonyms were grouped using Boolean operator *OR* and combined concepts using *AND*.

The search strategy included combinations such as: (healthcare student* OR medical student* OR health occupations student*) AND (“Health communication“[MeSH] OR interpersonal communication OR patient-provider communication) AND (immersive learning OR simulated learning OR immersive education OR immersive technologies OR educational technology) AND (“virtual reality“[MeSH] OR augmented reality OR mixed reality OR extended reality OR virtual patient). A detailed overview of the complete search strategy, including all search terms, database-specific adaptations, and applied filters, is provided in the Supplementary Materials.

The search was restricted using predefined filters for publication period, language (English), and publication type (systematic reviews, meta- analysis). All retrieved records were managed using Zotero (version 7.0 for Windows). In addition, forward and backward citation tracking of included systematic reviews was performed as a supplementary hand-search strategy [27]. Any publications identified through this process were required to meet the predefined eligibility criteria for inclusion.

### Title and abstract screening

A Microsoft Excel [28] spreadsheet was used to manage all identified articles. Two researchers independently conducted a pilot screening of titles and abstracts of a subset (20%) of the retrieved articles. The purpose of this pilot phase was to identify potential ambiguities, refine the screening criteria where necessary, and assess inter-rater reliability, in accordance with the Cochrane Handbook for Systematic Reviews of Interventions [29].

During this phase, researchers had access only to the titles and abstracts and were blinded to the full texts or each other’s assessments, ensuring independent and unbiased judgements. Inter-rater agreement for the pilot screening was calculated using Cohen’s kappa. Although the resulting kappa value of 0.66 indicated moderate agreement, the overall percentage agreement was very high (98.6%). This discrepancy can be explained by the so-called Cohen’s kappa paradox, in which high percentage agreement can coincide with only moderate kappa values when most decisions fall into the same category [30]. Given the high overall agreement and the satisfactory performance of the screening criteria, both researchers proceeded to independently screen all remaining titles and abstracts. Disagreements across all stages of study selection and synthesis were resolved through discussion; if consensus could not be reached, a third reviewer was consulted.

### Full text screening

**Fig. 1:**
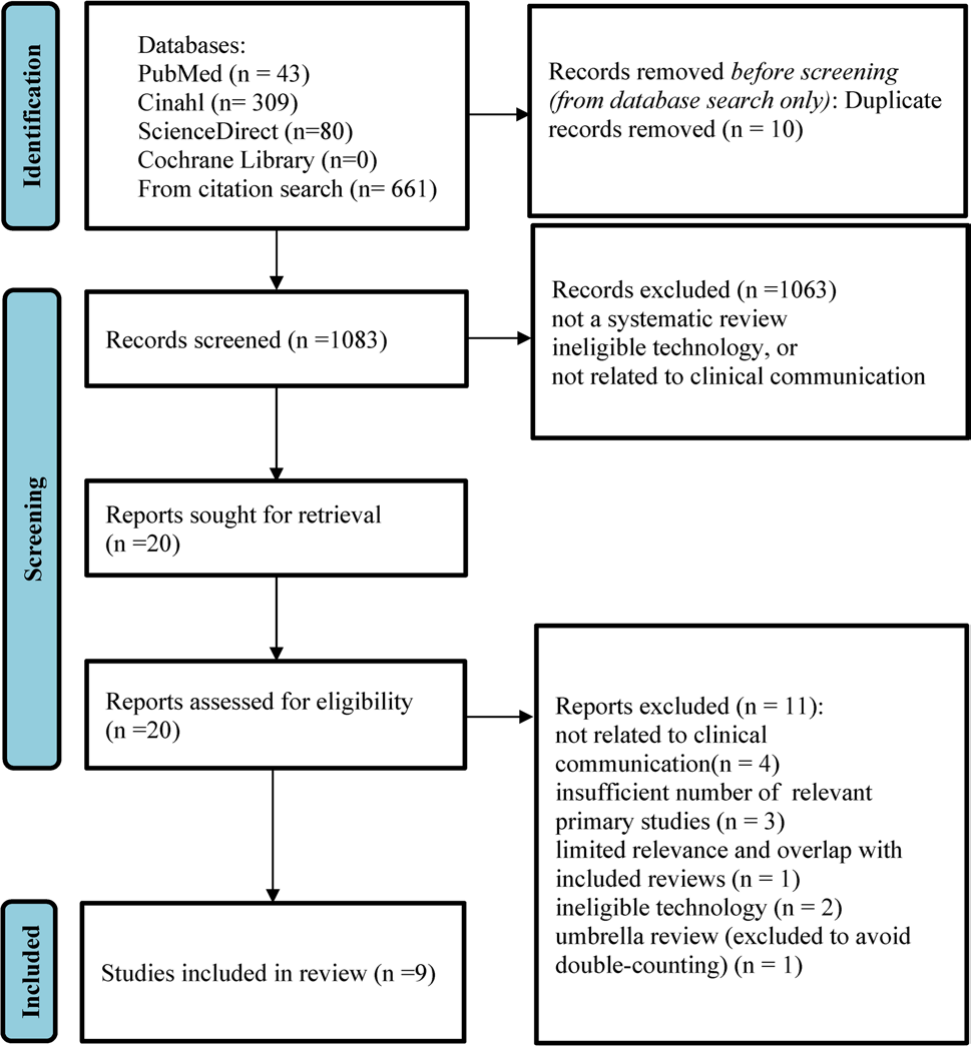
PRISMA 2020 flow diagram of study selection. Following pilot title and abstract screening, 17 conflicts (3.9%; including the update search) were resolved by consensus; during full-text screening, 2 conflicts (20%) were resolved by consensus

Two researchers independently retrieved and assessed the full texts of all potentially eligible articles using the predefined criteria. Blinding was not applied during the full-text screening phase, as detailed information on study characteristics (e.g., methodology, population, and interventions) was required to make informed eligibility decisions. The study selection process is summarized in the PRISMA 2020 flow diagram (Fig. 1) [23, 29].

### Data extraction

A standardized data extraction form was developed using Microsoft Excel [28]. For each included systematic review, the following data were extracted: study metadata (authors, year, journal, DOI, and database source); review characteristics (review type, number of included primary studies, and inclusion/exclusion criteria); sample characteristics (number and type of participants, profession, and demographics where available); intervention details (type of technology used for clinical communication training, learning modality, duration, and comparison condition); outcome measures (instruments used to assess clinical communication skills and reported primary and secondary outcomes); and reported effect sizes and statistical results (e.g., Cohen’s d, Pearson’s r, standardized mean differences, confidence intervals, and heterogeneity estimates). In addition, the methodological quality of each review was assessed using the AMSTAR-2 tool [31].

Data extraction was performed independently by two researchers. Extracted data were subsequently compared and discussed in a consensus meeting to resolve discrepancies.

To assess the risk of double-counting primary studies across included reviews, the Corrected Covered Area (CCA) was calculated according to Pieper et al. [32]. This metric quantifies the degree of overlap among primary studies and is recommended for use in umbrella reviews [32]. On this basis, umbrella reviews were excluded from the synthesis because their inclusion could have introduced duplicated evidence and increased the risk of double-counting [33].

### Methodological quality assessment

The methodological quality of the included reviews was assessed using the AMSTAR-2 tool [31]. The assessment was conducted by one researcher and cross-checked by a second researcher. Any discrepancies were resolved through discussion.

### Synthesis

Given the anticipated substantial heterogeneity across included reviews, a qualitative synthesis was conducted. Extracted data were organized according to intervention type, population characteristics, outcome measures, intervention setting, and comparison condition. To enhance reliability, data extraction and categorization were independently reviewed by two authors. Where appropriate, inductive codes were developed in addition to the predefined thematic categories. All categories were subsequently reviewed and refined through discussion.

Before finalizing the synthesis, both researchers cross-checked their interpretations to ensure consistency, transparency, and alignment with the extracted data. Data not reported in the original reviews were labeled as not reported (NR), and retracted reviews were excluded from the synthesis. Because all included reviews were rated as critically low in methodological quality, findings were interpreted cautiously rather than formally weighted by quality [31]. Owing to the absence of a meta-analysis, statistical assessment of publication bias was not performed. Instead, potential reporting bias was considered qualitatively, for example by examining whether reviews excluded grey literature or selectively emphasized positive outcomes.

Potential influences of methodological quality were explored narratively; however, meaningful comparisons across quality levels were not possible because all included reviews received uniformly critically low ratings. Accordingly, the purpose of this umbrella review was to identify patterns and recurring limitations in the review-level evidence base rather than to draw definitive conclusions about effectiveness.

## Results

The search strategy yielded 1,093 records, of which 10 duplicates were removed. Following title and abstract screening, 20 full-text articles were assessed for eligibility. After application of the inclusion criteria, nine systematic reviews were included in the qualitative synthesis (Fig. 1). Reasons for exclusion were categorized into five different aspects: (1) absence of clinical communication outcomes; (2) insufficient primary studies related to our research question; (3) use of non-eligible technologies regarding our inclusion criteria; (4) inclusion of a limited number of relevant primary studies with low coverage, which overlapped with primary studies identified in other included reviews; (5) umbrella reviews due to the risk of double counting (Supplementary Table S1. Full-text articles excluded with reasons).

Overlap of primary studies across the included systematic reviews was assessed using the Corrected Covered Area (CCA). The CCA was 0.9%, indicating only slight overlap and suggesting a minimal risk of double-counting within the synthesized evidence base [32].

### Overview of included reviews

We included eight systematic reviews, and one meta-analysis published between 2020 and 2025. The included reviews focused on undergraduate students across a range of healthcare disciplines, including pharmacy (*n* = 1571), medicine (*n* = 5359), nursing (*n* = 5170), psychology/psychiatry (*n* = 5904), and physiotherapy (*n* = 309). Learners were at different stages of undergraduate education, from first-year to the final-year students. Across the included reviews, most primary studies were conducted in the United States, followed by Australia and several European countries (e.g., the United Kingdom, the Netherlands, and Denmark), with a smaller proportion conducted in Asia and South America.

Across the included reviews, a wide range of immersive and VP-based technologies were examined. Most interventions involved low- to medium-immersion VP approaches delivered via web-based simulation platforms, interactive VP serious games, or VR-based simulations [18, 19, 34, 35]. High-immersion VR interventions additionally incorporated virtual ward environments and contextualized clinical settings [36]. Rodda et al. [37] included a heterogeneous set of immersive interventions, ranging from VR- and AR-based symptom simulations to virtual patient systems with predefined response formats, while one meta-analysis and one systematic review specifically focused on AR, VR, and metaverse-based simulations [20, 38]. One review included virtual simulation-based education combining 2D elements (e.g., photos, videos) and 3D-video, and sensory stimuli [39]. Interventions were implemented across diverse educational contexts, including university-based courses, clinical training environments, medical practices, and community pharmacy settings. Comparison conditions varied widely and included no intervention, traditional or alternative teaching methods, real patient encounters, non-VR simulation formats, or were not reported. In addition, several reviews included primary studies without a comparison group.

The included reviews examined immersive technologies across multiple clinical communication-related learning scenarios, including counseling encounters, clinical consultations, suicide and mental health risk assessments, routine clinical visits, and the delivery of unfavorable or sensitive information to patients and their families [18, 19, 34–37]. In addition, VR-based interventions were used to simulate patient perspectives associated with conditions such as dementia or schizophrenia [37].

**Fig. 2:**
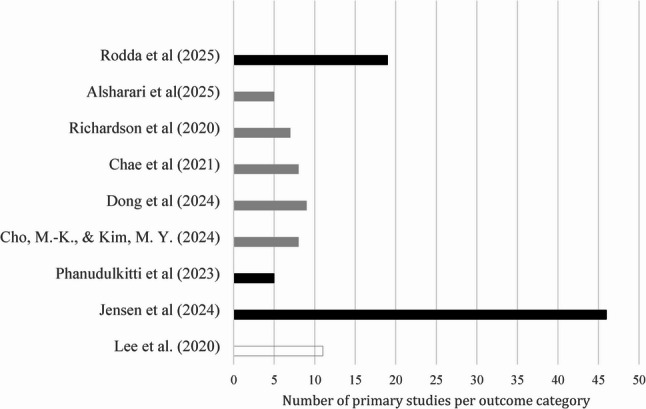
Harvest plot summarizing reported outcomes across included systematic reviews. Each bar represents a systematic review. Bar length reflects the number of communication-relevant primary studies within that review contributing to the predefined outcome category shown, because not all primary studies included in each review were relevant to the predefined outcomes of this umbrella review. Effect direction is indicated as follows: black = positive findings reported without comparison condition; grey = favoring the technology-based intervention; unfilled = mixed or inconsistent findings

A wide range of outcome measures were employed to assess communication-related learning outcomes. These included self-assessment instruments, course evaluations, and self-developed tools [18, 19, 34, 39]. Several reviews reported the use of Objective Structured Clinical Examinations (OSCEs) [40] and established communication scales (e.g., Empathic Communication Coding Scheme (ECCS)) [18, 19, 36–38]. Qualitative methods, including interviews and focus groups were also applied in some reviews [34, 36]. One review did not report specific outcome instruments and instead relied primarily on self-reported student perceptions and course evaluations [35]. An overview of study characteristics is provided in Supplementary Table S2, and a visual summary of outcome variability is presented in the harvest plot (Fig. 2).

Although only systematic reviews published within the past five years were included, the underlying primary studies covered a substantially broader time range, extending from 1993 to 2024. Several reviews included studies conducted before 2010, with some spanning more than three decades. This temporal heterogeneity is particularly relevant because the technological capabilities of virtual patient systems and immersive technologies, as well as learners’ familiarity with digital tools, have changed substantially over time.

### Risk of bias in studies

The methodological quality of the included systematic reviews was assessed using the AMSTAR-2 tool [31]. All reviews were rated as critically low in methodological quality, primarily because of recurrent limitations in key domains, including incomplete reporting of search strategies, lack of protocol registration, insufficient justification of excluded studies, and inadequate consideration of risk of bias when interpreting findings. These limitations should be considered when interpreting the results of this umbrella review. Detailed AMSTAR-2 item-level ratings are provided in Supplementary Table S3; item definitions follow the original AMSTAR-2 framework [31].

### Primary outcomes

For screen-based VPs, four reviews reported mixed findings on clinical communication outcomes. Lee et al. [19] described improvements in selected communication skills and clinical outcomes among medical students; however, non-verbal communication components were often insufficiently represented, and effects on empathy were weaker than in interactions involving real patients. Jensen et al. [18] reported advantages over no intervention or traditional teaching methods, but no clear superiority over non-technology-based simulations approaches; moreover, students frequently perceived VPs as lacking realism. Phanudulkitti et al. [35] reported favorable findings on pharmacy students’ confidence and consultation skills, although the absence of comparison conditions limits the interpretability of effectiveness. Richardson et al. [34] reported similar patterns in simulated pharmacy consultation settings, alongside persistent technical and usability barriers.

Across the included reviews, VR and AR interventions were more often associated with favorable findings than low-immersion approaches. Cho and Kim [20] identified a moderate effect in favor of VR/AR compared with traditional formats, although heterogeneity was high. Dong et al. [36] identified measurable improvements in clinical communication skills among nursing students but noted persistent limitations regarding the authenticity of patient interaction. Similarly, Rodda et al. [37] reported gains in learner engagement, attitudes, and perceived competence among medical and pharmacy students. However, technical limitations and usability challenges were reported consistently across reviews.

For simulation-based education with mixed levels of immersion, Alsharari et al. [38] reported improvements in communication skills and clinical competence among nursing students compared with conventional pre-clinical training formats, such as lecture-based instruction, skills-lab training, or standardized patient encounters. However, these comparison formats have themselves evolved substantially over time, and variability in study quality limited certainty of the evidence [14, 41]. Similarly, Chae et al. [39] reported favorable findings regarding empathy and cultural awareness among medical and healthcare students participating in virtual simulation.

### Secondary outcomes – barriers

For screen-based VPs, reported barriers included limited authenticity resulting from insufficient integration of non-verbal communication [19, 36], students’ perceptions of unrealistic interactions [18], high institutional resource requirements [35], and technical challenges such as software glitches and navigation difficulties [34].

For VR/AR and Metaverse-based interventions, scalability emerged as a key challenge. Cho and Kim [20] highlighted contextual barriers related to geographic and institutional variability, while Dong et al. [36] emphasized persistent concerns regarding the authenticity in VR-based teaching. Rodda et al. [37] reported heterogeneous student experiences, including usability issues, which negatively affected acceptance of immersive learning technologies.

In the context of mixed-immersion simulation-based education, Chae et al. [39] identified substantial heterogeneity in outcomes as a major research gap. Alsharari et al. [38] did not report specific implementations within the included primary studies. Collectively, these findings underscore the need for more systematic evaluation of implementation- and curriculum-related challenges across immersive learning approaches.

### Strength of evidence

Given the methodological limitations of the included reviews, the overall strength of evidence is limited, and the findings should be interpreted cautiously. Across reviews, VR/AR- and metaverse-based approaches were more frequently associated with favorable findings than screen-based VP approaches, whereas screen-based VP interventions yielded mixed findings and no clear advantage over non-technology-based simulation methods. Across all technology types, substantial heterogeneity in intervention design, outcome measures, and study populations limited comparability and confidence in the reported findings.

## Discussion

This umbrella review examined the extent to which immersive technologies and VP-based approaches have been reported to support the development of clinical communication skills in undergraduate healthcare education. Importantly, the primary contribution of this umbrella review is not to establish effectiveness, but to synthesize and critically appraise the current review-level evidence base. Accordingly, no definitive conclusions regarding effectiveness can be drawn.

Given the limitations of the current evidence base, immersive learning technologies and virtual patients are best positioned as supplementary components within a blended-learning structure rather than as stand-alone replacements for established clinical communication training formats. Their potential value appears greatest where they address needs that traditional methods meet only partially, for example in preparing students for sensitive communication encounters, enabling risk-free rehearsal before real patient contact, supporting perspective-taking experiences (e.g., dementia or mental health simulations), or providing repeated practice opportunities without requiring extensive clinical placement resources. Therefore, immersive technologies and virtual patient approaches should not be implemented as default innovations, but rather strategically in contexts where [1] learning objectives clearly match the affordances of the technology [2], educator support and feedback structures are in place, and [3] institutional resources allow for sustainable integration. At present, a measured adoption strategy appears most appropriate, using immersive tools as targeted enhancers of existing curricula rather than as replacements for established approaches.

Previous reviews have highlighted fragmented evidence, heterogeneous outcomes, and the absence of comprehensive synthesis across immersive learning interventions [15, 17, 21]. The present umbrella review supports these observations. Across the included reviews, interventions varied substantially in duration, instructional design, and curricular integration, and frequently lacked structured feedback mechanisms, all of which limit comparability and interpretability [18–20, 34–39].

In line with Kelly et al. [21], many interventions were implemented as isolated or single-session activities and were insufficiently embedded within broader curricular frameworks. Evidence regarding skill retention was inconsistent: whereas Jensen et al. [18] reported a lack of sustained effects, Richardson et al. [34] identified retention over several months, although this declined over time. Overall, the included reviews did not provide robust evidence on long-term outcomes or the transfer of communication skills into clinical practice [18, 19, 35].

Clinical communication is inherently multidimensional, encompassing verbal components (e.g., content structure and clarity of information), non-verbal elements (e.g., body language and facial expressions), and relational aspects such as empathy and rapport-building [3–5]. However, across the included reviews, these dimensions were inconsistently defined, operationalized, and measured. In particular, substantial challenges were evident in the assessment of non-verbal communication and empathy. Only a small number of studies explicitly addressed these dimensions, and those that did reported limited success in replicating the complexity and richness of real interpersonal interactions, which may help explain the limited and inconsistent findings regarding empathy and authentic interaction [19, 36].

From a technological perspective, immersive technologies and virtual patients differ substantially in terms of dialogue design, realism of verbal interaction, facial expression, movement, and contextual responsiveness. This distinction is important because the included reviews rarely differentiated clearly between levels of technological sophistication and generally provided limited detail on interaction quality, fidelity, or responsiveness. As a result, virtual patients and immersive systems were often discussed as relatively broad categories, which may obscure meaningful differences between technologies. This issue is particularly relevant in light of recent advances in artificial intelligence, especially large language models (LLMs), which have expanded the potential for more dynamic, context-sensitive, and naturalistic communication within virtual patient systems [42]. Consequently, the evidence synthesized in the included reviews may not fully reflect the capabilities of emerging systems, and the quality of interaction itself is likely to be an important determinant of communication skills acquisition [43].

As a result, the impact of immersive technologies and VPs on patient-related outcomes, such as satisfaction, adherence, or health-related improvements, remains largely unclear, underscoring the difficulty of capturing core elements of clinical communication, particularly empathy, within digital environments [3].

Across the included reviews, several pedagogical elements can be identified, although they were not systematically analyzed. Many interventions incorporated structured feedback (e.g., human-generated feedback systems, prebriefing, and debriefing), repeated practice opportunities, and simulation-based interaction that allowed learners to make decisions and experience consequences. These features are consistent with established pedagogical mechanisms such as deliberate practice, experiential learning, and reflective feedback processes [44–46]. This suggests that technological features and pedagogical design should not be considered independently, but as interacting components of communication skills training. However, because these elements were inconsistently reported and not systematically extracted, their specific contribution to learning outcomes remains unclear.

To further clarify this heterogeneity, immersive and virtual patient technologies can be differentiated along several key dimensions derived from the included reviews: (1) level of immersion (e.g., screen-based platforms vs. VR/AR simulations) (2), interaction design (e.g., predefined menu-based responses vs. natural language interaction), and (3) representational fidelity (e.g., static images, video-based responses, or fully animated 3D environments). The extracted data indicate substantial variation across these dimensions. Many virtual patient systems relied on predefined response options with limited interaction flexibility, whereas only a small number of studies incorporated typed or voice-based natural language input. Similarly, representations ranged from static or video-based patient responses to fully immersive 3D clinical environments. These differences are likely to influence learning outcomes, suggesting that interaction quality and feedback design, rather than technology alone, may represent key mechanisms underlying communication skills training [41]. These findings suggest that interaction quality and feedback design, rather than technology alone, may represent key mechanisms underlying effective communication skills training.

In addition, several reviews incorporated studies dating back to the 1990s, a period characterized by fundamentally different technological capabilities, including limited graphical fidelity, restricted interaction design, and lower user familiarity with digital systems. Consequently, findings from older virtual patient-based interventions may reflect technological and educational conditions that differ substantially from those of contemporary systems, particularly those integrating artificial intelligence and natural language processing.

Implementation-related barriers were also reported inconsistently and predominantly as secondary outcomes. Several reviews highlighted resource-related challenges such as institutional capacity, staffing requirements, and financial demands [19, 35, 36]. Additional obstacles included technical limitations, such as system glitches and navigation difficulties, which may negatively affect learner acceptance and feasibility [34].

Further heterogeneity arose from diverse study populations, varying curricular contexts, and inconsistent learning objectives across primary studies, as reported in the included reviews. Combined with the frequent use of non-standardized and non-validated outcome measures, these factors substantially limit comparability and constrain the ability to draw robust, generalizable conclusions.

Taken together, these findings highlight the need for greater methodological rigor in both primary studies and systematic reviews. Future research should prioritize the use of standardized and validated outcome measures, incorporate longitudinal follow-up designs, and apply methodologies capable of examining whether educational gains translate into patient-relevant outcomes. Mixed-method approaches and multilevel study designs, may be particularly valuable in capturing how changes in learners’ clinical communication skills influence patient satisfaction, trust, and other patient-centered outcomes [12, 47–51]. Such approaches align with Level 4 of Kirkpatrick’s evaluation model, which emphasizes behavioral change and improvements in patient outcomes [52].

### Limitations

This umbrella review has several limitations that should be considered when interpreting its findings. First, all included reviews were rated critically low in methodological quality, which limits confidence in the evidence synthesized. Second, pronounced heterogeneity was observed across the included reviews in terms of outcome measures, study populations, curricular contexts, and intervention designs. This variability restricts comparability and complicates the synthesis of consistent conclusions. Third, most reviews reported outcomes primarily in the short term, with little or no evidence regarding retention of clinical communication skills or their transfer into clinical practice.

An additional limitation of the current evidence base is the insufficient consideration of underlying pedagogical approaches and curricular integration. Across the included reviews, immersive technologies were often evaluated as stand-alone interventions, with limited reporting on instructional design principles, feedback structures, or alignment with curricular learning objectives. This lack of pedagogical contextualization limits interpretability, as learning outcomes in communication skills training are likely influenced not only by technological features, but also by didactic design. A clearer distinction between technological features (e.g., immersion and interactivity) and pedagogical design elements (e.g., feedback, scaffolding and alignment with learning objectives) may help future research to better isolate the active components of communication skills training.

As a result, conclusions regarding sustained educational impact remain limited. Fourth, barriers to curricular integration were reported inconsistently and were often addressed only as secondary outcomes. This limits insight into contextual, institutional and resource-related challenges that are critical for real-world adoption and scalability of immersive and VP-based interventions. Fifth, the literature search was restricted to peer-reviewed systematic reviews indexed in major databases and published in English. The exclusion of grey literature, preprints, and non-English publications may have contributed to publication bias and the omission of relevant evidence.

Given these limitations, conclusions should be interpreted conservatively. The primary contribution of this umbrella review lies in identifying overarching patterns and consistent research gaps within the field rather than in establishing definitive evidence of effectiveness.

## Conclusion

This umbrella review synthesizes systematic reviews examining immersive and virtual patient-based learning technologies for the development of clinical communication skills in undergraduate health professions education. Overall, the available review-level evidence suggests possible value in selected educational contexts, but remains insufficient to support firm conclusions regarding effectiveness. Findings should therefore be interpreted as preliminary and cautious indications rather than confirmatory evidence.

Future research should adhere to rigorous methodological standards, employ validated and standardized assessment instruments, and include longitudinal designs to evaluate the sustainability of learning effects and the transfer of communication skills into clinical practice. Greater emphasis should also be placed on curricular integration, structured feedback mechanisms, and resource considerations to support feasibility and scalability within educational programs.

At present, immersive and VP-based learning technologies should be viewed as adjunctive components within healthcare education rather than as stand-alone instructional approaches, as the available evidence does not justify replacing established teaching methods. High-quality primary studies and systematic reviews are needed to clarify under which conditions these technologies contribute meaningfully to the development of clinical communication skills.

## Supplementary Information

Supplementary Material 1.

Supplementary Material 2.

Supplementary Material 3.

Supplementary Material 4.

## Data Availability

All data generated or analyzed during this study are included in this published article and its supplementary information files.

## Abbreviations

AR: Augmented Reality
CCA: Corrected Covered Area
ECCS: Empathic Communication Coding Scheme
MR: Mixed Reality
OSCE: Objective Structured Clinical Examination
VP: Virtual Patient
VR: Virtual Reality
XR: Extended Reality
