# Supplementary material for: Teaching clinical communication skills through virtual patient-based learning: an umbrella review of systematic reviews
Source: BMC Med Educ. 2026 May 13;26:1082. doi: 10.1186/s12909-026-09374-6 (PMC13339519; doi:10.1186/s12909-026-09374-6)
Supplement: Supplementary file 2 — Supplementary Material 2. [file 12909_2026_9374_MOESM2_ESM.docx]

## **Terms and Filters**

**PubMed:** ("Students, Health Occupations"[MeSH] Healthcare Student* OR medical student* OR Health Occupations Student*OR pharmacy Student* OR psychiatry Student* OR psychology Student* OR nursing student* OR dietetic student* OR physiotherapy student* OR allied health student* OR health professional learner*) AND ("Health Communication"[MeSH] OR interpersonal communication OR patient-provider communication OR therapeutic communication OR health communication OR clinical communication skills OR counseling skills OR Health literacy OR soft skills OR patient* education OR patient*-centered communication) and (immersive learning OR simulated learning OR immersive education OR immersive technologies OR educational technology OR simulated environments OR simulation-based learning) AND ("Virtual Reality"[MeSH] OR augmented reality OR mixed reality OR extended reality OR virtual patient OR digital simulation)

Filter: December 31, 2019- December 31, 2024/ Meta-analysis, Systematic review

**Cinahl:** ("Healthcare student*" OR "medical student*" OR pharmacy student* OR “psychology Student*” OR psychiatry Student*” OR "nursing student*" OR "dietetic student*" OR "physiotherapy student*" OR "allied health student*" OR "health professional learner*" OR MH "Health Occupations Students" OR MH "Allied Health Personnel") AND ("interpersonal communication" OR "patient-provider communication" OR "therapeutic communication" OR "health communication" OR "clinical communication skills" OR "counseling skills" OR "Health literacy" OR "soft skills" OR "patient education" OR MH "patient-centered communication" OR MH "Communication" OR MH "Health Communication" OR MH "Professional-Patient Relations" OR MH "Patient Education") AND ("immersive learning" OR "simulated learning" OR "simulation-based learning" OR "immersive technologies" OR "simulated environments" OR MH "Simulation Training" OR MH "Educational Technology") AND ("virtual reality" OR "augmented reality" OR "mixed reality" OR "extended reality" OR "virtual patient" OR "digital simulation" OR MH "Virtual Reality" OR MH "Augmented Reality" OR MH "Computer Simulation")

Filter: December 31, 2019- December 31, 2024/peer reviewed/ English/ meta-analysis, systematic review

**ScienceDirect:** ("healthcare student" OR "medical student") AND (Communication OR education) AND ("immersive learning" OR "simulation-based learning") AND ("virtual reality" OR "augmented reality")

Filter: December 31, 2019- December 31, 2024/ Review articles/ English

**Cochrane Library:** ("Healthcare student*" OR "medical student*" OR “pharmacy student* OR psychology student* OR psychiatry student* OR "nursing student*" OR "dietetic student*" OR "physiotherapy student*" OR "allied health student*" OR "health professional learner*") AND ("interpersonal communication" OR "patient-provider communication" OR "therapeutic communication" OR "health communication" OR "clinical communication skills"OR "counseling skills" OR "Health literacy" OR "soft skills" OR "patient education" OR "patient-centered communication")AND ("immersive learning" OR "simulated learning" OR "simulation-based learning" OR "immersive technologies" OR "simulated environments" )AND ("virtual reality" OR "augmented reality" OR "mixed reality" OR "extended reality" OR "virtual patient" OR "digital simulation")

Filter: None
