# Supplementary material for: Teaching clinical communication skills through virtual patient-based learning: an umbrella review of systematic reviews
Source: BMC Med Educ. 2026 May 13;26:1082. doi: 10.1186/s12909-026-09374-6 (PMC13339519; doi:10.1186/s12909-026-09374-6)
Supplement: Supplementary file 4 — Supplementary Material 4. [file 12909_2026_9374_MOESM4_ESM.docx]

Table S3. AMSTAR 2 Item (1-16) evaluation of the included systematic reviews

| **Systematic Review** | **1** | **2** | **3** | **4** | **5** | **6** | **7** | **8** | **9** | **10** | **11** | **12** | **13** | **14** | **15** | **16** |
| --- | --- | --- | --- | --- | --- | --- | --- | --- | --- | --- | --- | --- | --- | --- | --- | --- |
| Lee et al. (19) | - | - | + | ± | + | + | - | - | - | + | Ø | Ø | - | + | Ø | + |
| Jensen et al. (18) | + | + | - | - | + | - | - | ± | - | - | Ø | Ø | - | - | Ø | + |
| Phanudulkitti et al. (35) | - | - | - | - | + | + | - | - | - | - | Ø | Ø | - | - | Ø | + |
| Cho & Kim (20) | + | + | + | ± | + | + | - | - | + | + | + | + | - | + | + | + |
| Dong et al. (36) | + | - | - | ± | + | + | - | - | - | - | Ø | Ø | - | - | Ø | - |
| Chae et al. (39) | + | - | + | ± | + | + | - | ± | - | - | Ø | Ø | - | + | Ø | + |
| Richardson et al. (34) | + | - | + | ± | - | - | - | - | - | - | Ø | Ø | - | - | Ø | + |
| Alsharari et al. (38) | - | - | - | - | + | + | - | ± | ± | - | Ø | Ø | - | + | Ø | + |
| Rodda et al. (37) | + | ± | - | - | + | + | + | ± | + | - | Ø | Ø | - | - | Ø | - |

Items 1–16 correspond to the AMSTAR-2 domains assessing methodological quality across protocol registration, search strategy, risk of bias, and synthesis methods (31).

**Note. AMSTAR-2 item-level ratings are shown for items 1–16.** + **= yes;** ± **= partial yes; - = no; Ø = not applicable. Two reviewers independently rated each review; disagreements were resolved by consensus (or third reviewer).**
